# Polish Medical Students’ Knowledge Regarding Human Papillomavirus’s Ways of Transmission, Risk of Cancer Development and Vaccination, and Their Intention to Recommend Vaccination

**DOI:** 10.3390/vaccines9070776

**Published:** 2021-07-12

**Authors:** Tomasz Milecki, Maciej Michalak, Jakub Milecki, Michał Michalak, Radosław Kadziszewski, Łukasz Kuncman, Piotr Jarzemski, Piotr Milecki, Andrzej Antczak

**Affiliations:** 1 Department of Urology, Poznan University of Medical Sciences, 61-701 Poznan, Poland; maciekmichalak@op.pl (M.M.); jakub.milecki@gmail.com (J.M.); radek_kadziszewski@o2.pl (R.K.); aa26@poczta.onet.pl (A.A.); 2 Department of Computer Sciences and Statistics, University of Medical Sciences, 61-701 Poznan, Poland; michal@ump.edu.pl; 3 Department of Radiotherapy, Medical University of Lodz, 90-419 Lodz, Poland; lukaszkuncman@gmail.com; 4 Department of Urology, Collegium Medicum in Bydgoszcz, Nicolaus Copernicus Univeristy in Torun, 87-100 Torun, Poland; piotr@jarzemski.pl; 5 Department of Electro–Radiology, University of Medical Sciences, 61-701 Poznan, Poland; piotr.milecki@wco.pl; 6 Radiotherapy Department, Greater Poland Cancer Centre, 61-866 Poznan, Poland

**Keywords:** HPV (human papillomavirus), medical education student, vaccination

## Abstract

Introduction: Human papillomavirus (HPV) is associated with six types of cancer in men and women. A vaccine against HPV, preferably administered before initial sexual intercourse, has been proven to be highly effective in preventing these cancers. An effective healthcare provider recommendation has significant influence on HPV vaccine uptake; therefore, it is critical that medical students receive comprehensive training in this area. Aim: The aim of the study was to assess the knowledge of medical students regarding Human Papillomavirus’s (HPV) ways of transmission, risk of cancer development, and vaccination against HPV. This study also investigated factors among medical students that would affect their intention to recommend HPV vaccination to others. Materials and Methods: The study was conducted among 1061 (678 women and 383 men) medical students who filled in our questionnaire. The medical students were divided into two subgroups: (1) pre-clinical medical students (MS pre-clinical; first-to third-year students; n = 683) and (2) clinical medical students (MS clinical; fourth-to six-year students; n = 378). Results: A total259 (24.41%) of the 1061 medical students were vaccinated against HPV. We found a significant improvement in the general level of knowledge in the later years of education (4–6) compared to the early years of education (1–3). However, it was demonstrated that, despite medical education advancements, there are still significant gaps of knowledge about the relationship between HPV infection and cancers other than cervical cancer, as well as in relation to the routes by which HPV is transmitted. Medical students’ intentions to recommend HPV vaccine to others were related to their own HPV-related knowledge and their own vaccination status. Conclusion: Medical students have gaps of knowledge regarding particular issues and aspects of HPV. It is necessary to further educate medical students in the field of prevention and in the treatment of lesions caused by HPV infection. Medical students’ intention to recommend the HPV vaccine can be improved by including them and members of their families in the HPV vaccination program.

## 1. Introduction

HPV has over 200 subtypes, the most dangerous of which are the oncogenic subtypes, whose current number is about 15 [1]. HPV is one of the most common sexually transmitted viruses [2]. According to epidemiological data, it is estimated that 70–80% of women are at risk of being exposed to HPV during their lives. Cervical cancer, one of the most common cancers in women, is closely related to HPV infection [3]. Gynecological scientific societies are encouraged to perform periodic check-ups, including cytology and HPV tests, as the gold standard in the secondary prevention of cervical cancer [4]. Moreover, as the primary prevention of cervical cancer, WHO recommends the implementation of HPV vaccination programs between the ages of 9 and 14, before becoming sexually active [5].

Initially, HPV vaccination programs were female-only programs. However, it has been demonstrated that HPV infection significantly increases the risk of development of other types of cancers, e.g., anal cancer, oropharyngeal cancer, laryngeal cancer, penile cancer, and vulvar cancer. It is estimated that 90% of cases of anal cancer are caused by HPV infection [6]. In the case of penile and vulvar cancer, this percentage is approximately 50% [7]. HPV infection is also currently the most common factor that increases the risk of developing oral cancer (60% of patients are infected with HPV), thereby outranking smoking, which has so far been positioned as the main risk factor [8]. Importantly, one meta-analysis showed that there is a high percentage of HPV infection among both women and men. This value is approximately 20% while, in the case of oncogenic variants of HPV, it is approximately 7.9% [9]. Therefore, the use of an HPV vaccine in men may also be justified from a medical point of view.

In particular regions of the world, HPV vaccination programs are recommended for both women and men. Particularly high vaccination efficacy rates of about 70% in men and 80% in women were obtained in Australia [10,11]. It is assumed that such a strategy should significantly reduce the spread of HPV in society, which in turn should yield measurable effects in terms of reducing the incidence of the cancers referred to above. Poland is one of the few countries in the European Union where a national HPV vaccination program has not been implemented, and the current availability of the vaccine is limited only to commercial centers and local programs. The coexistence of a number of factors, such as an economic barrier, a lack of social awareness, and social and ideological influences, affects the current situation in Poland [12].

Increasing HPV vaccination rates is in the interests of society. Achieving this goal requires a multi-pronged measures, including conducting campaigns and educational programs for society and developing a proper training system for healthcare professionals, including students of medical universities [13]. Therefore, it is necessary to periodically evaluate the state of knowledge about HPV and vaccination, as well as awareness and individual preferences among students at medical universities and healthcare professionals. Such information may be further used to correct any irregularities which may occur. The number of scientific reports on this subject in relation to the Polish education and healthcare systems is still limited; therefore, we decided to create an up-to-date assessment of the state of knowledge on HPV and HPV vaccination preferences among medical students.

The aim of the study was to assess the knowledge of medical students regarding Human Papillomavirus’s (HPV) ways of transmission, the risk of cancer development, and vaccination. This study also investigated factors among medical students that would affect their intention to recommend HPV vaccination to others.

## 2. Material and Methods

### 2.1. Material

The study was conducted among 1061 (678 women and 383 men) medical students at Karol Marcinkowski Poznan University of Medical Sciences between November 2019 and December 2019 (Table 1). After analysis of the medical education curriculum for HPV (Supplementary Material File S1), the medical students were divided into two subgroups: (1) pre-clinical medical students (MS pre-clinical; first-to third-year students; n =  683) and (2) clinical medical students (MS clinical; fourth-to six-year students; n =  378). Only, 35.25% (n = 239) of female medical students and 5.22% (n = 20) of male medical students were vaccinated against HPV (24.41% of all medical students were vaccinated against HPV, n = 259). The mean age of first sexual intercourse among the surveyed students was 18.42 ± 2.00 years.

### 2.2. Questionnaire

A self-designed questionnaire (32 questions) was created after analysis of the available literature. The first part of questionnaire contained nine questions about basic characteristics and personal sexual history. The second part of the questionnaire included fifteen questions concerning knowledge of HPV and HPV vaccination. The internal consistency of the questionnaire assessing knowledge on HPV was performed by Cronbach’s alfa coefficient. For our HPV knowledge questionnaire, Cronbach α level was 0.70. The last part of the questionnaire contained eight questions about HPV awareness and attitudes towards HPV vaccination.

A pilot study was carried out among 20 medical students of the Poznań University of Medical Sciences in order to test and evaluate the questionnaire in terms of clarity and completeness of the questions asked. After taking into account the suggestions of the students participating in the pilot study and introducing the suggested changes, the proper study was launched. The questionnaires were distributed to the students in classrooms at the beginning of lectures. The participants were informed as to the purpose of the study. Each participant provided written consent to participate in the study. Time to fill in the questionnaire was unlimited, but it usually took no more than 10 min.

### 2.3. Statistical Analysis

Each participant in the study scored 1 point for giving a correct answer, and no points were awarded or deducted for an incorrect answer or the answer “I do not know”. Students were compared in terms of the point numbers and the percentage of correct answers they obtained by completing the questionnaire. The levels of knowledge were described by median, mean, standard deviation, and percentage of correct answers. Student’s t-tests were used to compare the two groups. A comparison of the percentages of responses between the analyzed groups was made using a test of proportions.

A logistic regression model, both univariate and multivariate, was used to analyze the relationships between specific factors (demographic data, personal sexual history, level of knowledge about HPV, personal vaccination status against HPV, respondent’s immediate family vaccination status against HPV) and the recommendation of HPV vaccination among medical students. The results of the analysis are presented as an odds ratio (OR) and a 95% confidence interval (95% CI). For the multivariate logistic regression model, a stepwise backward selection was used.

The calculations were made with the use of TIBCO Software Inc. (2017) Statistica (3307 Hillview Ave, Palo Alto, CA, USA), version 13. http://statistica.io, accessed on 20 April 2020. All tests were analyzed at the significance level of *p* = 0.05.

The estimated sample size of 310 students was required for a 5% margin of error and a 95% confidence interval (CI). A total of 1155 students participated in our survey. Finally, 1061 of them who fully completed the questionnaire were included in the study. Incomplete or incorrectly filled in questionnaires were eliminated from the analysis. The number of questionnaires is therefore sufficient to assess the level of knowledge in particular groups of students and to find statistically significant differences between them.

## 3. Results

### 3.1. Level of Knowledge

The mean score (total level of knowledge) among all the respondents was 11.74 ± 2.51 points out of a maximum of 15 points (Table 1). First of all, we found that several personal sexual history data of medical student had an impact on total knowledge levels regarding HPV. Students who had sexual intercourse in the past had a higher level of knowledge than students who had not experienced sexual intercourse (*p* < 0.001). Moreover, heterosexual students had a lower level of knowledge than homosexual/bisexual students (*p* < 0.001). Furthermore, students with a permanent sexual partner showed a higher level of knowledge than students without a sexual partner (*p* < 0.001). A higher number of sexual partners in the past was also associated with a higher level of knowledge (*p* < 0.001).

Our study also demonstrated an improvement in HPV knowledge among medical students who were more advanced in their education. Students of higher years of study (4–6) had a higher level of knowledge about HPV and HPV vaccination than students in their early years of study (1–3) (*p* < 0.001) (Table 2). First of all, along with advancement in education, we observed a significant general improvement in the state of knowledge in the field of HPV cancer risk development (questions 1–4) (*p* < 0.001), as well as in the field of genital warts (question-5) (*p* < 0.001) and in the field of differential diagnosis with the Herpes Virus (question-6) (*p* < 0.001). Medical students (both early and late years) showed significantly less awareness of the HPV-related risks of cancer development in locations other than the cervix, such as the anus, the penis, and the vulva. Moreover, our study demonstrated that medical students in later years of study had a significantly higher level of knowledge about the possible routes of HPV transmission than those in their early years of study (questions 12 and 13) (*p* < 0.001). In the question about vertical routes of transmission as a source of HPV infection (question 14), students in years 1–3 were more likely to give the correct answer that vertical transmission is associated with a potential risk of HPV infection than students in years 4–6 (*p* < 0.001); however, both groups of medical students had a low level of knowledge in this matter. Finally, we found that in relation to the question about the recommended age for HPV vaccination (question 15), students in later years of study gave the correct answer more often (*p* < 0.001).

### 3.2. Intention to Recommend HPV Vaccination

Table 3 represents the results of univariate and multivariate logistic regression, which demonstrated the impact of specific factors (personal sexual history, level of knowledge about HPV, personal vaccination status against HPV, respondent’s immediate family vaccination status against HPV) on the opinion regarding the mandatory implementation of an HPV vaccination, as well as on the intention to recommend HPV vaccination to others (women and men separately). Overall, 79% (n = 839) of all medical students had the opinion that HPV vaccination should be mandatory, while 21% (n = 222) of the medical students were against such an idea. The final multivariate logistic regression model demonstrated that higher levels of knowledge about HPV (OR = 1.12, 95%CI: 1.04–1.20) and a positive HPV vaccination status by a member of the respondent’s immediate family (OR = 3.12, 95%CI: 1.70–5.75) were both factors that significantly supported their opinion about mandatory vaccination against HPV.

Our study also demonstrated that, among medical students, there is a significant preference for recommending vaccination to women as opposed to men (86% vs. 59% *p* < 0.001) (Table 4). Overall, 86% (n = 911) of the medical students would recommend HPV vaccination to women, while 14% (n = 150) were opposed to this. The final multivariate logistic regression model demonstrated that a higher level of knowledge about HPV (OR = 1.13, 95%CI: 1.01–1.27), positive vaccination status against HPV (OR = 3.89, 95%CI: 1.62–9.32), and HPV vaccination by a member of the respondent’s immediate family (OR = 3.22, 95%CI: 1.33–7.79) were significant factors among medical students who supported recommending an HPV vaccination to women. Overall, 59% (n = 628) of the medical students would recommend HPV vaccination to men, while 41% (n = 433) said they would not do so (Table 5). Among medical students, it was demonstrated that positive vaccination status against HPV (OR = 1.95, 95%CI: 1.27–3.02), positive vaccination status against HPV by a member of the respondent’s immediate family (OR = 1.55, 95%CI: 1.01–2.38), higher year of study (OR = 1.14, 95%CI: 1.01–1.28), and higher level of knowledge about HPV (OR = 1.10, 95%CI: 1.04–1.20) were all significant factors that supported opinions regarding the recommendation of an HPV vaccination to men.

## 4. Discussion

The study performed by our research team is an analysis that assesses the state of knowledge about HPV and preferences in terms of HPV vaccination among Polish medical students with respect to sex, degree of education, sexual data, and experience with HPV vaccination (respondent was vaccinated or a member of the respondent’s immediate family was vaccinated).

Our analysis has shown a significant improvement in the level of knowledge about HPV among students, along with progress in the educational process. Moreover, it was demonstrated that students who have already experienced sexual intercourse and students who have had more sexual partners in the past scored better in the survey. The above results are consistent with the data obtained by the WHO, which assume, above all, a positive effect in terms of public health and an increase in awareness among the public (i.e., also students and healthcare professionals) based on prevention measures, including information and education, implemented on a global scale [5].

One of the few sources we can rely on is a study from a culturally distant part of the world, i.e., China, in which 1878 medical students were examined. This was a multi-center study, which included six selected universities from south-western China [14]. The study referred to above demonstrated that the knowledge about HPV is at the level of 76.5%, which is a result similar to that obtained in our study. The study demonstrated a low level of knowledge about effective prophylaxis, i.e., vaccination among Chinese medical students. Only 48% of them knew about the existence of such a vaccine and knew the recommendations in terms of indications for use. However, detailed knowledge of HPV-induced diseases and their treatment was at a significantly lower level compared to the results obtained by our research team. Less than 43% of male students and 49% of female students were able to answer 10 or more questions out of 22 questions that were included in the standardized research sheet. One of the common conclusions that emerges from the comparison of the two studies is the significant increase in knowledge about HPV, along with progress in medical education, among the study participants (OR: 1.39; 95% CI: 1.11–1.75). In another recently published study, low levels of knowledge about HPV among young Polish doctors have been reported. As many as 34.7% of physicians obtained a result from questionnaire in the lowest quadrille (54–59% of correct answers) [15]. A worrying finding is that the lowest level of knowledge is found among pediatric doctors who have contact with the target group for vaccination programs (children aged 9–14 years).

In the general population, even in countries where educational programs on the prevention of HPV infection have been implemented, the level of knowledge is insufficient and, surprisingly, has deteriorated over the last decade [16]. The lowest awareness of the subject was observed in men, racial minorities, inhabitants of rural areas, people with a lower socioeconomic status and lower level of education, and the elderly. The numerical results of our study are difficult to compare to studies carried out in the general population due to the different complexities of the questionnaires.

Another issue raised by our research team was the assessment of the state of knowledge on the relationship between HPV infection and increased risk of developing particular types of cancer. We observed a significant general improvement in the state of knowledge in this field, along with progress of the educational process but, in the case of the analysis of individual cancers, there were some significant differences that require separate commentary. First of all, it should be emphasized that almost all students in both their early and late years of education are highly aware that HPV infection increases the risk of cervical cancer. Our analysis also shows that students in their early years of study are significantly less aware of the increased risk of developing cancers in other locations. Importantly, we observed that the system of training in the medical program contributes to a significant improvement in the knowledge of medical students, particularly with regard to head and neck cancer (from 63.10% to 94.71%, *p* < 0.001). However, about 20% of medical students in their final years of study are not aware of the relationship between HPV infection and the increased risk of developing cancer in other locations, i.e., the penis and anus. These results are reflected in studies conducted by other research teams, which indicate a lack of knowledge among medical students in this field [15,17,18].

Another important observation concerns the relatively low percentage of women vaccinated against HPV in the studied population (35% of all female medical students). Moreover, the HPV vaccination coverage of Polish teenagers is even lower, and is estimated to be 7.5–10% [12]. In Poland, HPV vaccination was implemented in a national immunization program in 2007; however, it is not funded by public resources [19]. The importance of HPV vaccination in Poland is emphasized by numerous medical societies, which provide particular guidelines (based on worldwide recommendations) for healthcare practitioners. For example, the Polish Gynecological Society recommend HPV vaccinations among girls aged between 11–12, and agree on so-called supplementary vaccination in girls 13 to 18 years old [20]. Guidelines from the Polish Pediatric Society also emphasize that vaccinations in girls older than 11-12 years of age should be conducted before sexual initiation [21]. Similarly, the Polish Society of HPV Infections Prophylaxis (PTPZ-HPV) recommends preventive vaccination in girls aged 12 to 15 [22].

Polish medical guidelines also provide recommendations for conducting HPV vaccination in young boys aged 9–15 [22]. This issue is also raised by the Polish Urological Association, which respects European Urological Guidelines (EAU), where a statement of early prophylactic HPV vaccination in boys before the onset of sexual activity is strongly recommended [23]. In our study, all medical students showed a significant preference for recommending vaccinations to women as opposed to men (85.86% vs. 59.19%). Moreover, we found that only 5.22% of all male medical students were vaccinated against HPV. The problem of HPV vaccination among men is even present in countries in which preventive vaccination programs for both sexes have been introduced [24]. Undoubtedly, both society and healthcare professionals mainly associate HPV vaccine with cervical cancer and, therefore, in public opinion, HPV vaccine is regarded as a vaccine for women [25]. This results in the misconception that only women benefit from the vaccine, which translates into a lower percentage of vaccinated men.

An important issue that has been raised by our research team concerns the factors that predict the attitude of medical students when it comes to recommending HPV vaccination. The vast majority of respondents who participated in our study would recommend HPV vaccination, and most of them believe that HPV vaccination should be mandatory. In our multifactorial analysis, we have shown that medical students were more likely to recommend HPV vaccination to both women and men if the following criteria were met: they had higher level of knowledge about HPV, they were vaccinated against HPV, or a member of the respondent’s immediate family was vaccinated against HPV. These results are consistent with the results of other studies, where a better general level of knowledge about HPV and positive HPV vaccination status have been shown to be positive predictive factors [26,27]. These results indicate that engaging medical students (as well as members of their families) to take up the HPV vaccination may improve their attitudes regarding recommending HPV vaccination to others. However, medical students, who will become future healthcare professionals, should also have an appropriate level of understanding regarding HPV, the vaccine, and its protection coverage. Taking into consideration our aforementioned findings, we suggest that medical students’ attitudes for recommending HPV vaccine to others may be increased by improving their level of knowledge, especially in terms of the links between HPV-related infections and other types of cancer.

Finally, our study found that medical students also have gaps in their knowledge about HPV protection methods and HPV transmission routes. More than 90% of all respondents are aware of the fact that condoms contribute to reducing the risk of HPV infection. However, one in five medical students in their early years of education are not aware that HPV infection is possible during anal or oral sex. Moreover, only half of the students are aware that the HPV virus may also be transmitted vertically. This shows that medical students do not have full knowledge of this important matter. These results are confirmed in other research papers, which show, for example, that a significant proportion of respondents are wrongly convinced that the use of condoms provides fully effective protection against HPV infection [28].

It has to be emphasized that our study has several limitations. First of all, the data were collected in only one region of Poland and the medical students attended only one medical school. The HPV-related curriculum provided by the university where the study was performed mostly concerned the virus’s molecular and epidemiological aspects. Undoubtedly, the provided curriculum also does not include training in more practical aspects such as methods of recommendation HPV vaccination to others. Finally, the study was performed using a researcher-designed questionnaire; however, there is no validated questionnaire in this area.

## 5. Conclusions

Increasing HPV vaccination rates in society has proven to be a cost-effective intervention that prevents HPV infection and various types of cancer. Educational programs and a proper training system for healthcare professionals, including students at medical universities, are crucial for achieving this goal. We demonstrated that, despite medical education advancement, there are still significant knowledge gaps about HPV. Our survey showed data that can be useful in the design of future HPV programs and services, as they highlight gaps in the education (especially in the area of HPV-related types of cancer) of future medical professionals. Finally, we also demonstrated that the attitudes of medical students when it comes to recommending HPV vaccination to others can be improved by encouraging them, as well as members of their families, to take up HPV vaccination.

## Figures and Tables

**Table 1:** Characteristics and personal sexual history of participants in the study and their association with HPV knowledge.

Variable	Knowledge of HPV					
	**Number (% of All)**	**Median Score**	**Mean Score**	**% of Correct Answers**	**SD**	***p*-Value**
**Year of study**						
1–3	683 (64%)	11	10.88	72.53%	2.51	<0.001
4–6	378 (36%)	14	13.29	88.60%	1.34	
**Sex**						
Female	678 (63%)	12	11.69	77.93%	2.48	0.8479
Male	383 (37%)	12	11.81	78.73%	2.44	
**Sexual intercourse in the past**						
Yes	741 (70%)	13	12.01	80.07%	2.31	<0.001
No	320 (30%)	12	11.11	74.07%	2.49	
**Sexual orientation**						
Heterosexual	984 (93%)	12	11.67	77.80%	2.50	0.006
Other than heterosexual	77 (7%)	13	12.60	84.00%	2.00	
**Permanent sexual partner**						
Yes	495 (47%)	13	12.09	80.60%	2.25	<0.001
No	566 (53%)	12	11.43	76.20%	2.46	
**Number of sexual partners in the past**						
0–2	857 (81%)	12	11.61	77.40%	2.48	<0.001
3+	204 (19%)	13	12.26	81.73%	1.98	
**HPV vaccination**						
Yes	259 (24%)	12	11.35	75.67%	2.45	0.086
No	802 (76%)	13	11.86	79.07%	2.42	

**Table 2:** Comparison of percentages of correct responses to particular questions provided by students in early years of education (1–3) and late years of education (4–6).

	Question	1–3 (683 Students)	4–6 (378 Students)	*p*-Value
**1.**	Does HPV infection increase the risk of developing cervical cancer?	669 (98%)	377 (99%)	<0.05
**2.**	Does HPV infection increase the risk of developing penile and vulvar cancer?	347 (51%)	304 (80%)	<0.001
**3.**	Does HPV infection increase the risk of developing oropharyngeal cancer, oral cancer and laryngeal cancer?	431 (63%)	358 (95%)	<0.001
**4.**	Does HPV infection increase the risk of developing anal cancer?	327 (48%)	312 (83%)	<0.001
**5.**	Does HPV infection increase the risk of genital warts?	440 (64%)	366 (97%)	<0.001
**6.**	Can HPV infection cause genital herpes?	225 (33%)	288 (77%)	<0.001
**7.**	Does the number of sexual partners affect the risk of HPV infection?	656 (96%)	375 (99%)	<0.001
**8.**	Do condoms reduce the risk of HPV infection?	632 (93%)	354 (94%)	0.576
**9.**	Is it possible to cure HPV?	357 (52%)	269 (71%)	<0.001
**10.**	Can you be HPV-positive and have no symptoms?	636 (93%)	375 (99%)	<0.001
**11.**	Can you contract HPV through sexual intercourse?	678 (99%)	378 (100%)	0.096
**12.**	Can you contract HPV through anal sex?	542 (79%)	366 (97%)	<0.001
**13.**	Can you contract HPV through oral sex?	528 (77%)	365 (97%)	<0.001
**14.**	Is vertical transmission of HPV possible?	450 (66%)	189 (50%)	<0.001
**15.**	What is the recommended age for HPV vaccination?	513 (75%)	346 (92%)	<0.001

**Table 3:** Factors influencing medical students’ opinions about mandatory HPV vaccination (univariate and multivariate model).

Should HPV Vaccination Be Mandatory? YES n = 839 (79.08%), NO n = 222 (20.92%)						
**Logistic Regression**	**Univariate Model**			**Multivariate Model**		
**Variable:**	**OR**	**95% CI**	***p*-Value**	**OR**	**95% CI**	***p*-Value**
**Sex (ref.-men)**	1.43	1.05–1.93	0.02	1.41	0.96–2.06	0.074
**Year of study (ref.-first year) ** **(for each one year of education)**	1.19	1.09–1.31	0.000			
**Sexual intercourse in the past (ref.-no)**	1.55	1.14–2.12	0.005			
**First sexual intercourse (ref.-<18 y.o.)**	1.21	0.83–1.76	0.317			
**Sexual orientation (ref. heterosexual)**						
**Homosexual**	3.96	0.93–16.72	0.061		NA	
**Bisexual**	1.12	0.53–2.36	0.761			
**Permanent sexual partner (ref.-no)**	1.27	0.94–1.72	0.110			
**Number of sexual partners (ref. 0–2)**						
**>2**	1.46	0.97–2.20	0.065			
**Vaccinated against HPV (ref.-no)**	2.11	1.41–3.15	0.000			
**Member of immediate family vaccinated against HPV (ref.-no)**	2.65	1.68–4.16	0.000	3.12	1.70–5.75	0.000
**Level of knowledge (for each one point)**	1.13	1.07–1.20	0.000	1.12	1.04–1.20	0.002

NA—not applicable in final model.

**Table 4:** Factors influencing medical students’ intention to recommend HPV vaccination to women (univariate and multivariate model).

Would You Recommend HPV Vaccination to a Woman? YES n = 911 (85.86%), NO n = 150 (14.14%)						
**Logistic Regression**	** Univariate Model **			** Multivariate Model **		
**Variable:**	** OR **	** 95% CI **	*** p * -Value **	** OR **	** 95% CI **	*** p * -Value **
**Sex (ref.-men)**	0.99	0.69–1.42	0.978		NA	
**Year of study (ref.-first year)** **(for each one year of education)**	1.26	1.12–1.41	0.000	1.19	0.98–1.44	0.073
**Sexual intercourse in the past**	1.78	1.24–2.54	0.001			
**First sexual intercourse ** **(ref. <18 y.o.)**	0.88	0.56–1.38	0.586			
**Sexual orientation** **(ref. heterosexual)**					NA	

**Homosexual**	1.12	0.38–3.24	0.835			
**Bisexual**	1.10	0.46–2.65	0.822			
**Permanent sexual partner**	1.61	1.13–2.30	0.009			
**Number of sexual partners**						
**(ref. 0–2)**						
**>2**	2.56	1.44–4.53	0.001	1.76	0.94–3.28	0.076
**Vaccinated against HPV (ref.–no)**	5.92	2.97–11.8	0.000	3.89	1.62–9.32	0.002
**Member of immediate family ** **vaccinated against HPV**	4.95	2.48–9.90	0.000	3.22	1.33–7.79	0.009
**Level of knowledge ** **(for each one point)**	1.18	1.11–1.26	0.000	1.13	1.01–1.27	0.024

NA—not applicable in final model.

**Table 5:** Factors influencing medical students’ intention to recommend HPV vaccination men (univariate and multivariate model).

Would You Recommend HPV Vaccination to a Man? YES n = 628 (59.19%), NO n = 433 (40.81%)						
**Logistic Regression**	** Univariate Model **			** Multivariate Model **		
**Variable:**	** OR **	** 95% CI **	***p*-Value **	** OR **	** 95% CI **	***p*-Value **
**Sex (ref.-men)**	0.99	0.77–1.28	0.968	0.71	0.51–1.00	0.05
**Year of study (ref.-first year) (for each one year of education)**	1.16	1.07–1.25	0.000	1.14	1.01–1.28	0.022
**Sexual intercourse in the past (ref.-no)**	1.37	1.05–1.79	0.018			
**First sexual intercourse ** **(ref. <18 y.o.)**	0.91	0.67–1.23	0.553			
**Sexual orientation**						
**(ref. heterosexual)**						
**Homosexual**	2.46	1.05–5.78	0.037		NA	
**Bisexual**	1.64	0.87–3.12	0.127			
**Permanent sexual partner (ref.-no)**	1.17	0.91–1.49	0.210			
**Number of sexual partners**						
**(ref. 0–2)**						
**>2**	1.65	1.19–2.29	0.002			
**Vaccinated against HPV (ref.-no)**	1.71	1.27–2.31	0.000	1.95	1.27–3.02	0.002
**Member of immediate family vaccinated against HPV (ref.-no)**	1.69	1.24–2.32	0.001	1.55	1.01–2.38	0.043
**Level of knowledge ** **(for each one point)**	1.12	1.07–1.17	0.000	1.10	1.00–1.17	0.035

NA—not applicable in final model.

## Data Availability

The data that support the findings of this study are available from the corresponding author, [T.M.], upon reasonable request.

## Supplementary Materials

Supplementary Material File S1 “The curriculum for HPV in medicine at the University Karol Marcinkowski Poznan University od Medical Sciences” is available online https://www.mdpi.com/article/10.3390/vaccines9070776/s1.
