# Supplementary material for: Polish Medical Students’ Knowledge Regarding Human Papillomavirus’s Ways of Transmission, Risk of Cancer Development and Vaccination, and Their Intention to Recommend Vaccination
Source: Vaccines (Basel). 2021 Jul 12;9(7):776. doi: 10.3390/vaccines9070776 (PMC8310167; doi:10.3390/vaccines9070776)
Supplement: Supplementary file 1 [file vaccines-09-00776-s001.zip › vaccines-1200202-supplementary.pdf]

Supplementary Material File S1 The curriculum for HPV in medicine at the University Karol Marcinkowski Poznan University od Medical Sciences is spread over 6 years of study.

First year–third year (pre-clinical years of education):
Cytophysiology—the mechanism of action of oncogenic viruses in neoplastic transformation on example of HPV 16.
Microbiology—information about the structure of HPV and types of viruses.

Fourth year–sixth year (clinical years of education):
Epidemiology—information on risk factors and preventive measures.
Pathomorphology-basic knowledge about HPV, laboratory diagnostics, cytological, and histological examinations.
Dermatology—causes, symptoms, principles of diagnosis, and therapeutic management of sexually transmitted diseases.
Gynecology—prevention of genital cancers and principles of their treatment in the course of HPV infection. Physical and physical examination in gynecology and gynecological oncology.
Mastering theoretical knowledge about the human papillomavirus, etiopathogenesis, diagnosis and treatment, including the basics of gynecological oncology.
Prevention, diagnosis, and epidemiology of cervical cancer.

Oncology—prophylaxis, epidemiology, risk factors in human papillomavirus infection. Effect of oncogenic viruses on head and neck cancers. Getting to know the principles of functioning of screening programs in Poland.

Infectious diseases—HPV types, routes of infection, HPV-related diseases, HPV infection prophylaxis.
